# Relationship between cognitive function in individuals with diabetic foot ulcer and mortality

**DOI:** 10.1186/s13098-022-00901-1

**Published:** 2022-09-19

**Authors:** Yael Sela, Keren Grinberg, Tali Cukierman-Yaffe, Rachel Natovich

**Affiliations:** 1 Nursing Department, Faculty of Social and Community Sciences, Ruppin Academic Center, Emeq-Hefer, Israel; 2 Endocrinology Division, The Center for Successful Aging with Diabetes, The Gertner Institute for Epidemiology and Health Policy Research, Sheba Medical Center, Ramat Gan, Israel; 3 Epidemiology Department, Sackler Faculty of Medicine, The Herczeg Institute on Aging, Tel-Aviv University, Tel Aviv, Israel; 4 The Rehabilitation Hospital, Sheba Medical Center, Ramat-Gan, Israel

**Keywords:** Diabetes mellitus, Mortality, Cognitive dysfunction, Diabetic foot ulcer, Cognitive tests

## Abstract

**Background:**

Diabetic foot ulcer (DFU) is a common diabetes mellitus (DM) complication. Individuals with DM and a DFU achieved significantly lower scores in cognitive tests than those without a DFU. We investigated whether baseline cognitive function in individuals with a DFU is a determinant of mortality.

**Methods:**

A prospective study using data collected during a case–control study conducted in 2010–2012 whereby 90 participants with a DFU (mean age at baseline 58.28 ± 6.95 years, 75.6% male) took the paper and pencil and the NeuroTrax battery of cognitive tests. Depression was assessed, and the DFU status was evaluated. In 2020, information pertaining to participants’ vital status (dead/alive) was collected and the relationship between baseline cognitive status and vital status was assessed.

**Results:**

During a median follow-up of 6.8 years (range 0.2–9.5), 39 participants died (43.3%). Individuals alive vs. those who had died during follow-up had a higher global cognitive score at baseline (92.16 ± 10.95 vs. 87.18 ± 12.24, p = 0.045), but increased risk was not found. Individuals who were alive vs. those who had died during follow-up had statistically significantly higher baseline executive function, reaction time and digit symbol substitution test results. However, after adjustment for glycosylated hemoglobin (HbA1c), microvascular and macrovascular complications, no relationship between cognitive tests and mortality remained significant.

**Conclusions:**

The higher mortality rate among people with type 2 DM and a DFU was not significant after adjustment for HbA1c, micro- and macrovascular complications. There may be common pathophysiological pathways to both DM complications and cognitive impairment, which may contribute to increased mortality. Further studies are warranted.

**Supplementary Information:**

The online version contains supplementary material available at 10.1186/s13098-022-00901-1.

## Introduction

Diabetes mellitus (DM) affects both small and large blood vessels [1]. Most DM complications are related to vascular disease, and are classified as microvascular (neuropathy, nephropathy and retinopathy) and macrovascular (peripheral arterial disease, cardiovascular and cerebrovascular disease) [2]. One of the most common complications of DM is diabetic foot, which is defined by the International Working Group on the Diabetic Foot (IWGDF) as “an infection, ulceration, or destruction of tissues of the foot of a person with currently or previously diagnosed diabetes mellitus, usually accompanied by neuropathy and/or peripheral artery disease in the lower extremity” [3]. Diabetic foot manifests in deep tissue lesions or ulceration, which can lead to skin infection, ulcer formation and even destruction of deep tissues that can eventually bring to the loss of a limb. This complication has a global prevalence of 6.3% and a lifetime incidence of up to 25% [4]. It is a leading cause of hospitalization, morbidity and mortality among people with DM.

Diabetic foot ulcer (DFU) is usually a consequence of several factors, of which the main contributing factors are neuropathy, vascular disease and limited joint mobility [5]. Both sensory and autonomic neuropathy, as well as certain foot deformities and a greater body mass, independently influence the risk of DFU [6]. The outcome of DFU depends on ulcer-related factors (e.g., infection, necrosis and/or gangrene), the presence of peripheral arterial disease, as well as on the patient’s age and co-morbidities that may influence wound healing [7].

Several studies have shown that patients with a DFU have more than two-fold increase in mortality compared to patients with DM who do not have a DFU, regardless of other risk factors [8–11].

DM is also associated with changes in cognition; several epidemiological studies have suggested that it contributes to the development of cognitive dysfunction [12]. Increasing evidence suggests that microvascular dysfunction is also one of the major underlying mechanisms for cognitive impairment [13, 14]. Several studies have shown that dysregulation of cerebrovascular integrity and function, such as reduced cerebral blood flow and higher blood brain barrier permeability, exacerbates neurovascular damage after ischemic injury and impair vasotrophic coupling necessary for repair processes, thereby leading to the development of neuronal pathologies and cognitive impairment [15–20]. Cognitive function declines more rapidly in individuals with DM, and they have a 1.5-fold higher risk of cognitive decline, and a 1.6-fold higher risk of future dementia compared to those without DM [12]. Autopsies of 2365 individuals showed that diabetes plus infarcts was associated with lower cognitive scores at end of life than pathology of infarcts or diabetes alone, suggesting that DM increases the risk of cerebrovascular pathology [21]. A cross-sectional study has demonstrated that diabetic retinopathy increased the risk of multiple microbleeds in the brain by twofold; over time, retinopathy together with microbleeds increased the risk of dementia [22]. In a study that evaluated 180 patients with type 2 DM, glycosylated hemoglobin (HbA1c) levels, insulin therapy and diabetic retinopathy were associated with cognitive impairment after coronary artery bypass grafting [23]. Qiu et al. showed that DM is related to poor performance on cognitive tests of executive function and information-processing speed. This relationship was mainly mediated by markers of neurodegeneration and cerebrovascular disease [24]. Lower scores in cognitive tests have been associated with brain atrophy, which underlies poor cognitive performance predicting dementia and death [25]. In an analysis of 2977 individuals with type 2 DM who were middle-aged and older, an inverse relationship was observed between cognitive function (measured by digit symbol substitution test [DSST]) and incident cardiovascular events (non-fatal myocardial infarct, non-fatal stroke, or death from cardiovascular causes) [26].

Our group has previously shown that individuals with a DFU had significantly lower cognitive scores in all tested cognitive domains compared to individuals with DM without this complication [27]. Tuttolomondo et al. have also reported that individuals with a DFU had lower mean Mini Mental State Examination scores compared to people with DM who did not have this complication [28]. Brognara et al. showed that lower cognitive function scores using a generalized measure of basic cognitive proficiency, psychomotor processing speed, executive function and scanning task were related to cutaneous changes and foot disorders in patients with DM ≥ 65 years of age [29]. In contrast, Siru et al. did not find differences in cognition between patients with type 2 DM and a DFU and patients without a DFU from community or hospital-based clinics—even after adjustment for age, sex, education, diabetes duration, and random blood glucose [30].

It is not known whether DFU is a surrogate marker for more advanced micro- and macro-vascular complications of DM, which also underly cognitive impairment, or if this condition’s inflammatory outcomes independently contribute to mortality [31]. Impaired cognition may also be associated with impaired self-care, risk of falls, risk of medication errors and increased frailty—all of which may contribute to the development and deterioration of DFU. Furthermore, there is little data on the relationship between DFU and mortality due to specific causes. Therefore, the purpose of this study was to assess whether impaired cognitive function in people with a DFU is a determinant of mortality.

## Methods

### Participants and setting

This follow-up study used data collected during a case–control study conducted in 2010–2012 that examined whether the cognitive profile of people with DM and a DFU differs from that of people with DM without this complication. Information on participants’ vital status (dead/alive) was collected and combined with the information collected in the original study. The inclusion/exclusion criteria for the case–control study have been previously described [27]. Briefly, 99 individuals aged 45–75 years with type 2 DM and a diagnosis of a DFU complication were recruited from diabetic foot clinics and orthopedic departments in two hospitals. Exclusion criteria included illiteracy, significant hearing or visual disability, a diagnosis of dementia or cognitive impairment that could impair the individual’s ability to provide informed consent, a history of stroke, liver disease, renal failure with serum creatinine above 2.0 mg/dL, liver disease, a history of stroke, or any major non-diabetes-related illness expected to reduce the participant's life expectancy within two years or to interfere with study participation.

The study was approved by Sheba Medical Center’s ethics committee and all participants signed an informed consent prior to participation in the study.

### Data collection

Baseline data related to disease severity, adequacy of treatment and possible covariates and mediators were collected during the case–control phase of the study. The patients completed a questionnaire with demographic and medical information. The following data were collected from the participants' medical files: diabetes duration (defined as the time from diagnosis), smoking status, insulin treatment, number of hypoglycemia events, hypertension (defined as either reported hypertension or hypertension lowering medication use or systolic blood pressure > 140 mmHg/diastolic blood pressure > 90 mmHg), dyslipidemia (defined either as reported dyslipidemia or statin use or low-density lipoproteins > 100 mg/dl), the number of diabetes microvascular complications per participant (defined as retinopathy, nephropathy [serum creatinine, microalbumin-creatinine ratio], neuropathy), the number of diabetes macrovascular complications per participant (defined as ischemic heart disease including angina and myocardial infarction, peripheral vascular disease), HbA1c levels, creatinine levels, and body mass index. DFU was evaluated at baseline using the University of Texas San Antonio (UTSA) system.

Cognitive function was assessed using the NeuroTrax computerized cognitive assessment battery of tests, designed for early detection of mild cognitive impairment and mild dementia. The results of this battery of tests were analyzed for the following measures: (1) a global cognitive score (GCS), which is the mean of the cognitive domains examined (excluding nonverbal intelligence quotient); (2) a nonverbal intelligence quotient, which entailed solving visual tasks; (3) scores in five specific cognitive domains: attention and concentration, memory, psychomotor efficiency, reaction time, and executive function.

In addition, the following paper and pencil cognitive tests were used: (1) DSST—a subtest of the Wechsler Adult Intelligence Scale—that includes an array of cognitive domains (e.g., short-term memory, concentration, attention, capacity for learning and visual motor speed and coordination); and (2) a verbal fluency test measuring language, semantic memory and verbal production [32].

The Patient Health Questionnaire (PHQ-9) was used for evaluating depression [33].

### Assessment of outcome: vital status

In 2020 the participants’ vital status (dead or alive) for the period 9 October 2011 to 4 November 2019 was collected from patient medical records and from the Israeli Ministry for the Interior’s registry.

### Statistical analysis

Statistical analysis was conducted using SAS version 9.4 (SAS, Institute Inc., Cary, NC, USA). The distribution of baseline characteristics among those alive and dead at follow-up was calculated. The χ^2^ test was used for comparing categorical variables, and the t-test—for continuous variables. Univariable logistic regression was performed for each of the cognitive function tests. Multivariable stepwise logistic regression was performed for each of the cognitive function tests that were significant in the univariate analysis, with HbA1c entered in the first step, HbA1c and microvascular complications entered in the second step and HbA1c, microvascular complications and macrovascular complications entered in the third step. A p-value less than 0.05 with a bi-directional hypothesis was considered statistically significant.

## Results

At follow-up information on vital status was available for 90 participants. The participants’ demographic and clinical characteristics at baseline are summarized in Table 1. The mean age of the study population at baseline was 58.3 ± 7.0 years and most participants (75.6%) were male. During a median follow-up of 6.8 years (range, 0.2–9.5), 39 participants died (43.3%). There was no difference in baseline characteristics between the group of 90 participants that were analyzed in the current study and the 9 participants who were lost to follow-up after the original study (Additional file 1: Table S1).

**Table 1:** Baseline demographic and clinical characteristics of the study population

Variable	Total N = 90	Alive N = 51	Dead N = 39	P value^f^
Sex				
Male	68 (75.6)	39 (76.5)	29 (74.4)	0.817
Female	22 (24.4)	12 (23.5)	10 (25.6)	
Age (years)	58.3 ± 7.0	56.4 ± 6.8	60.8 ± 6.3	0.002
Years of Education	12.5 ± 3.0	12.8 ± 3.3	12.6 ± 2.9	0.754
Diabetes duration^a^ (years)	15.2 ± 8.0	13.9 ± 7.6	17.0 ± 8.2	0.075
Dyslipidemia^b^	70 (77.8)	38 (74.5)	32 (82.1)	0.394
Hypertension^c^	72 (80.0)	37 (72.5)	35 (89.7)	0.043
Active smoker	21 (23.3)	16 (31.4)	5 (12.8)	0.039
Ischemic heart disease	40 (44.4)	15 (29.4)	25 (64.1)	0.001
Peripheral vascular disease	64 (71.1)	34 (66.7)	30 (76.9)	0.287
Glycosylated hemoglobin (%)	8.8 ± 2.1	8.6 ± 2.0	9.2 ± 2.4	0.231
Insulin use	72 (80.0)	41 (80.4)	31 (79.5)	0.915
Use of statins	65 (72.2)	34 (66.7)	31 (79.5)	0.178
Use of aspirin	60 (66.7)	28 (54.9)	32 (82.1)	0.007
Retinopathy	47 (52.2)	22 (43.1)	25 (64.1)	0.049
Nephropathy	34 (37.8)	9 (17.6)	25 (64.1)	< .0001
Neuropathy	80 (88.9)	45 (88.2)	35 (89.7)	0.822
Creatinine (mg/dL)	1.2 ± 0.7	1.1 ± 0.9	1.3 ± 0.4	0.202
Body mass index (kg/m^2^)	30.1 ± 6.1	30.3 ± 5.8	29.9 ± 6.6	0.748
Low-density lipoproteins (mg/dL)	74.4 ± 31.8	76.6 ± 34.7	71.6 ± 27.9	0.470
Hypoglycemia events with need for medical attention	0.9 ± 2.4	1.2 ± 3.0	0.5 ± 1.3	0.179
Hypoglycemia events treated by patient	2.6 ± 1.4	2.6 ± 1.4	2.6 ± 1.4	0.810
Diabetic foot UTSA score	9.1 ± 4.1	8.8 ± 4.5	10.41 ± 3.5	0.063
PHQ9 Depression score	5.9 ± 6.0	5.3 ± 5.8	6.8 ± 6.1	0.241
Diabetes-related microvascular^d^ complications	1.8 ± 0.9	1.5 ± 0.8	2.2 ± 0.9	0.0002
Diabetes-related macrovascular^e^ complications	2.2 ± 1.3	1.8 ± 1.1	2.8 ± 1.3	0.0003

Categorical variables are shown as number and percentage and continuous variables are shown as mean ± standard deviation

^a^ Defined as time since diagnosis; ^b^ Defined as reported dyslipidemia/statin use/low-density lipoproteins > 100 mg/dl; ^c^ defined as reported hypertension/hypertension lowering medication use/systolic blood pressure > 140 mmHg/diastolic blood pressure > 90 mmHg; ^d^ Number of complications per participant, defined as retinopathy/nephropathy/neuropathy; ^e^ Number of complications per participant, defined as myocardial infarction/angina/peripheral vascular disease; ^f^P value by χ2 test for categorical variables or by Student’s t-test for continuous variables

The participants who were still alive at follow-up were significantly younger on average than the participants who had died (56.4 ± 6.8 vs. 60.8 ± 6.3 years, p = 0.002). No other statistically significant differences in demographic parameters were observed between the participants who had died and those who were alive at follow-up. Among participants who had died vs. those alive at follow-up there was a higher rate of hypertension (89.7% vs. 72.5%, p = 0.043), cardiovascular disease (64.1% vs. 29.4%, p = 0.001), nephropathy (64.1% vs. 17.6%), microvascular complications (2.18 ± 0.94 vs. 1.49 ± 0.76, p = 0.0002) and macrovascular complications (2.8 ± 1.3 vs. 1.8 ± 1.1, p = 0.0003). Interestingly, there was a significantly greater rate of smokers in the group of participants that were still alive (31.4% vs. 12.8%, p = 0.039). The mean severity of DFU was lower in the group of participants who were alive at follow-up compared to the group of participants who had died, however, the difference between the groups was not statistically significant (8.8 ± 4.5 vs. 10.4 ± 3.5, p = 0.063).

Comparison of baseline characteristics showed no statistically significant difference between individuals who had a GCS below median and those who had a GCS above median, except for age (60.0 ± 7.1 vs. 56.6 ± 6.5, p = 0.019, Table 2).

**Table 2:** Comparison of baseline demographic and clinical characteristics by Neurotrax global cognitive score

	Neurotrax global cognitive score		
	Above median N = 45	Below median N = 45	P value^b^
Sex			
Male	37 (82.2)	31 (68.9)	0.141
Female	8 (17.8)	14 (31.1)	
Age (years)	56.6 ± 6.5	60.0 ± 7.1	0.019
Years of education	13.0 ± 3.2	12.0 ± 2.6	0.107
Highest degree			
Elementary school	8 (17.8)	7 (15.6)	0.398
High school	17 (37.8)	27 (60.0)	
Non-academic higher education	5 (11.1)	3 (6.6)	
Academic education	15 (33.3)	8 (17.8)	
Marital status			
Single	3 (6.7)	3 (6.7)	0.676
Married	31 (68.9)	35 (77.8)	
Divorced	8 (17.8)	6 (13.3)	
Widowed	3 (6.7)	1 (2.2)	
Employment status			
Employed	23 (51.1)	15 (33.3)	0.082
Retired	4 (8.9)	12 (26.7)	
Volunteer work	1 (2.2)	0 (0.0)	
Unemployed	17 (37.8)	18 (40.0)	
Diabetes duration^a^ (years)	15.2 ± 7.4	15.3 ± 8.6	0.927
Glycosylated hemoglobin (A1C%)	8.9± 2.0	8.8 ± 2.3	0.725
Glycosylated hemoglobin (mmol/mol)	74 ± NA^c^	73 ± NA^c^	0.725

Categorical variables are shown as number and percentage and continuous variables are shown as mean ± standard deviation

^a^ Defined as time since diagnosis; ^b^ P value by χ2 test for categorical variables or by Student’s t-test for continuous variables; ^c^ Standard deviation could not be converted to mmol/mol because NGSP HbA1c is below 3%

### Relationship between cognitive function and mortality in individuals with DF

Individuals alive vs. those who had died during follow-up had a higher GCS at baseline (92.16 ± 10.95 vs. 87.18 ± 12.24, p = 0.045), but the odds ratio (OR) did not reach statistical significance (OR = 0.96 [95% CI 0.93–1.00], p = 0.050). Individuals who were alive at follow-up has statistically significant higher baseline executive function, reaction time and DSST compared to individuals who had died during follow-up (Table 3).

**Table 3:** Participant cognitive baseline characteristics and outcome

	Total Mean ± SD	Alive N = 51 Mean ± SD	Dead N = 39 Mean ± SD	P value	Odds Ratio	(95% CI)	P-value
NeuroTrax scores^a^							
Global Cognitive Score	90.00 ± 11.72	92.16 ± 10.95	87.18 ± 12.24	0.045	0.96	(0.93–1.00)	0.050
Memory	90.00 ± 14.41	91.43 ± 13.15	88.14 ± 15.89	0.286	0.98	(0.96–1.01)	0.283
Executive Function	91.32 ± 12.66	94.02 ± 13.11	87.79 ± 11.25	0.020	0.96	(0.92–0.99)	0.024
Attention	89.94 ± 15.30	91.95 ± 15.44	87.31 ± 14.91	0.155	0.98	(0.95–1.01)	0.157
Motor Skills	89.84 ± 17.41	91.54 ± 16.89	87.47 ± 18.08	0.288	0.99	(0.96–1.01)	0.286
Reaction time	91.47	94.62	87.40 ± 14.92	0.015	0.96	(0.93–0.99)	0.019
Non-Verbal IQ (Problem solving)	96.84 ± 14.27	98.19 ± 14.97	95.08 ± 13.28	0.309	0.98	(0.96–1.01)	0.305
Digit symbol substitution test^c^	6.06 ± 3.01	6.78 ±3.25	5.13 ± 2.41	0.009	0.81	(0.68–0.96)	0.014
Verbal Fluency-Phonemic^b^	− 2 ± 1	− 2 ± 1	− 2 ± 1	0.579	0.92	(0.68–1.24)	0.576
Verbal Fluency-Semantic^b^	− 1 ± 1	− 1 ± 1	− 1 ± 1	0.897	0.97	(0.66–1.44)	0.896

*CI* confidence interval, *SD* standard deviation

^a^ Adjusted for age and years of education; ^b^ standardized for age, presented as Z-scores; ^c^ Standardized for age

Every 1 unit increase in executive function, reaction time, and DSST score was associated with a lower risk of death (OR = 0.96 [95% CI 0.92, 0.99; p = 0.024], 0.96 [95% CI 0.93, 0.99, p = 0.019], and 0.81 [95% CI 0.68, 0.96; p = 0.014], respectively), (Table 3).

After adjustment for HbA1c, microvascular and macrovascular complications, none of the relationships between the cognitive tests and mortality remained significant.

## Discussion

Of 90 individuals with DFU who had participated in the original study and had available vital status, 43% had died during the follow-up period. Those who had died during follow-up had statistically significantly lower executive function and reaction time scores in the Neurotrax battery of tests, and statistically significantly lower DSST compared to the participants who were still alive at follow-up. Test scores were standardized for age, reducing the possibility that the observed difference arises from differences in age. In addition, the participants who had died during the follow-up period had higher rates of micro and macrovascular disease compared to those who are still alive. After adjusting for HbA1c, micro, and macrovascular disease, no association between cognitive impairment and mortality was observed.

Several studies have shown that individuals with a DFU have excess risk for all-cause mortality than those with diabetes only [9, 34–36]. In a meta-analysis of 11 studies that reported 84,131 deaths from any cause in 446,916 individuals with DM, those with a DFU had a 2.45 times higher pooled relative risk of all-cause mortality compared to those without this complication, which was attributed to greater event rates of fatal cardiovascular and cerebrovascular disease [35]. The results of our study, which showed a 43% mortality rate among individuals with a DFU after a mean of 6.8 years of follow-up are in line with other studies that showed approximately 40% mortality in individuals with DM and a DFU after 5–10 years of follow-up [36–38]. Other studies have demonstrated that cognitive impairment also confers a higher mortality risk among individuals with DM [39–41].

Microvascular disease might be the common underlying process which causes cognitive dysfunction in people with DM and mediates the development of DFU. Neuropathy, vascular disease and limited joint mobility are major contributing factors to the development of DFU [6]. Marseglia et al. showed that among patients with diabetic foot aged 65 and over, episodic memory impairment was associated with microvascular complications (OR 9.68) and foot amputation (OR 4.13) [42].

Our analysis also suggests that HbA1c is a contributing factor to mortality in patients with a DFU and cognitive impairment. Among hospitalized patients with a DFU, the duration of glycemic control was found to be inversely associated with greater risk for amputation and all-cause mortality [43]. In another study, higher HbA1c was associated with reduced cognitive scores of patients with diabetes and lacunar stroke [44]. In contrast, glycemic control did not prevent cognitive decline among males and females with type 2 DM who participated in the ACCORD-MIND study [45] and in the Veterans Affairs Diabetes Trial [46]; however, in the ACCORD study glycemic control delayed the onset of albuminuria and some measures of eye complications and neuropathy [47]. Another study has shown that elderly patients (≥ 65 years) with HbA1c < 7% (53 mmol/mol) had increased odds for psychomotor slowness (OR 7.75) and abstract reasoning impairment (OR 4.49) [42].

Excess mortality in patients with reduced cognitive performance and a DFU may be related to the severity of underlying vascular complications. In addition, the decline in executive function in individuals with DM can complicate the management of the disease, especially in patients with a DFU, creating a vicious cycle. Evidence-based guidance for managing and preventing diabetic foot, which was published by the IWGDF and the American Diabetes Association, underlined the importance of educating patients on self-care practices for preventing ulcers and their recurrence [48, 49]. However, these recommendations require using complex cognitive abilities to learn, understand, and remember new information, plan and initiate self-care practices, adopt behavioral changes that involve psychomotor skills and maintain these behaviors while controlling and suppressing impulses. Indeed, self-care and cognition are closely inter-connected. Self-management in DM is affected by specific cognitive functions, such as immediate memory, attention, visual-spatial/constructional capabilities, and specific executive functions, such as problem solving and planning. In a study performed among 1,398 older adults with DM who were living in the community, increased cognitive impairment reduced participants’ adherence to each DM-related self-care task with incremental growth in DM comorbidity [50]. Cognitive functioning also predicted the success of rehabilitation programs following amputation [51]. Poor mental status was negatively associated with functional mobility, falls, adherence to therapy, use of prostheses and their fit, and maintaining pre-operative independence after amputation [52]. Therefore, interventions to reduce cardiovascular and cerebrovascular risk may reduce mortality. Young et al. showed that an aggressive program of cardiovascular risk management can reduce mortality rates to as low as 26% in individuals with a DFU [53].

The limitations of the study include its relatively small sample, which prevented us from fully addressing possible confounding variables (other than age and years of education to which the cognitive test scores were already adjusted). All patients had a DFU at baseline and there were no differences in baseline parameters, such as HbA1c levels, between the groups analyzed. However, additional changes, such as changes in glycemic control, that may have occurred during the years of follow-up, may have affected mortality. Indeed, cognitive impairment leads to lower self-care and inadequate diabetes management, such as glycemic control. Additionally, the cause of death was not analyzed. The strength of the study lies in the comprehensive cognitive tests that the participants performed at study baseline.

## Conclusions

The study showed a higher mortality rate among people with type 2 DM and a DFU, which was not significant after adjustment for HbA1c, micro- and macrovascular complications. There may be common pathophysiological pathways to DM complications and cognitive impairment which may contribute to increased mortality. Although further studies are required, routine cognitive testing in older people with DM could target patients who are at a higher risk for complications and mortality.

## Supplementary Information

**Additional file 1: Table S1.** Comparison of baseline demographic characteristics between participants analyzed in the study and participants who were lost to follow-up.

## Data Availability

The datasets used and/or analyzed during the current study are available from the corresponding author on reasonable request.

## Abbreviations

DFU: Diabetic foot ulcer
DSST: Digit symbol substitution test
DM: Diabetes mellitus
GCS: Global cognitive score
HbA1c: Glycosylated hemoglobin
IWGDF: International Working Group on the Diabetic Foot
OR: Odds ratio
PHQ-9: Patient Health Questionnaire
