# Supplementary material for: Relationship between cognitive function in individuals with diabetic foot ulcer and mortality
Source: Diabetol Metab Syndr. 2022 Sep 19;14:133. doi: 10.1186/s13098-022-00901-1 (PMC9487125; doi:10.1186/s13098-022-00901-1)
Supplement: Supplementary file 1 — Additional file 1: Table S1. Comparison of baseline demographic characteristics between participants analyzed in the study and participants who were lost to follow-up. [file 13098_2022_901_MOESM1_ESM.docx]

**Table S1. Comparison of baseline demographic characteristics between participants analyzed in the study and participants who were lost to follow-up**

| **Variable** | **Total**  **N=90** | | **Lost to follow-up N=9** | | **P value^f^** |
| --- | --- | --- | --- | --- | --- |
| Sex |  |  |  |  |  |
| Male | 68 | (75.6) | 8 | (88.9) | 0.367 |
| Female | 22 | (24.4) | 1 | (11.1) |  |
| Age (years) | 58.3 | ±7.0 | 55.7 | ±5.8 | 0.279 |
| Years of Education | 12.5 | ±3.0 | 11.4 | ±1.9 | 0.317 |

Categorical variables are shown as number and percentage and continuous variables are shown as mean ± standard deviation
